# Everolimus Nanoformulation in Biological Nanoparticles Increases Drug Responsiveness in Resistant and Low-Responsive Breast Cancer Cell Lines

**DOI:** 10.3390/pharmaceutics11080384

**Published:** 2019-08-02

**Authors:** Arianna Bonizzi, Marta Truffi, Marta Sevieri, Raffaele Allevi, Leopoldo Sitia, Roberta Ottria, Luca Sorrentino, Cristina Sottani, Sara Negri, Elena Grignani, Serena Mazzucchelli, Fabio Corsi

**Affiliations:** 1 Nanomedicine Laboratory, Department of Biomedical and Clinical Sciences “Luigi Sacco”, Università di Milano, 20157 Milano, Italy; 2 Environmental Research Center, ICS MAUGERI SPA SB, Institute of Pavia, IRCCS, 27100 Pavia, Italy; 3 Breast Unit, Istituti Clinici Scientifici Maugeri IRCCS, 27100 Pavia, Italy

**Keywords:** breast cancer, Everolimus, nanoparticles, H-ferritin

## Abstract

Everolimus (Eve) is an FDA approved drug that inhibits mammalian target of rapamycin (mTOR). It is employed in breast cancer treatment even if its responsiveness is controversial. In an attempt to increase Eve effectiveness, we have developed a novel Eve nanoformulation exploiting H-ferritin nanocages (HEve) to improve its subcellular delivery. We took advantage of the natural tumor targeting of H-Ferritin, which is mediated by the transferrin receptor-1 (TfR1). Breast cancer cells overexpressing TfR-1 were successfully recognized by H-Ferritin, displaying quick nanocage internalization. HEve has been tested and compared to Eve for in vitro efficacy in sensitive and resistant breast cancer cells. Nanoformulated Eve induced remarkable antiproliferative activity in vitro, making even resistant cell lines sensitive to Eve. Moreover, the antiproliferative activity of HEve is fully in accordance with cytotoxicity observed by cell death assay. Furthermore, the significant increase in anticancer efficacy displayed in HEve-treated samples is due to the improved drug accumulation, as demonstrated by UHPLC-MS/MS quantifications. Our findings suggest that optimizing Eve subcellular delivery, thanks to nanoformulation, determines its improved antitumor activity in a panel of Eve-sensitive or resistant breast cancer cell lines.

## 1. Introduction

Advanced breast cancer (BC) still represents a clinical challenge due to the high rate of chemoresistance and consequent cancer progression, both in hormone receptor-positive and negative subtypes [1]. Different drivers of resistance have been described for HER2-positive, triple-negative, and hormone receptor-positive cancers. Intriguingly, a common shared feature has been identified downstream in the proliferative pathway of BC cells: the hyperactivation of the phosphatidylinositol 3-kinase (PI3K)/AKT/mammalian target of rapamycin (mTOR) pathway irrespectively from the growth factor receptors status [2]. The PI3K/AKT/mTOR pathway can display different pathological alterations, such as the downregulation of the tumor suppressor PTEN [3,4,5], the abnormal activation of PI3K [6,7,8], or the overexpression/hyperactivation of AKT [9]. Everolimus (Eve; RAD001, Afinitor^®^) is an FDA-approved rapamycin analog that acts inside the cytoplasmatic compartment, inhibiting the PI3K/AKT/mTOR pathway. Specifically, Eve inhibits mTOR, interrupting its signaling cascade and resulting in inhibition of cell proliferation and growth [10]. Although Eve is currently approved for second-line treatment of metastatic BC, sometimes showing encouraging results, conflicting clinical data have been reported on its efficacy, particularly in some hormone receptor-positive and in triple-negative BC. Furthermore, a relatively small advantage in progression-free survival is severely paid by patients with the well-known Eve-related toxicities, mainly stomatitis, affecting the quality of life [1,2].

Nanotechnology has offered new opportunities to overcome current major challenges of chemotherapy through biological targeted nanosystems [11,12]. In particular, H-ferritin (HFn) nanocages hold a great promise thanks to their specific tumor homing mediated by Transferrin Receptor-1 (TfR1) internalization [13,14]. HFn is a 12 nm cave sphere constituted by 24 H-subunits of ferritin protein. H-monomers have been produced in *E. coli* by DNA recombinant technology and they self-assemble into their spherical quaternary structure after protein synthesis. Moreover, from the nanotechnological point of view, they combine low toxicity with high stability in biological fluids and with the possibility to load different kind of molecules inside its cavity [14,15,16,17,18,19]. Therefore, several HFn-based nanodrugs have been developed, obtaining specific tumor recognition, improved drug penetration, and increased activity with reduced side-effects due to toxicity. Furthermore, it has been demonstrated that nanoformulation in HFn has a role in avoiding the onset of chemoresistance [20]. Despite this, the potential of HFn as nanovector to deliver drug inside the cytoplasm has not been well investigated and only indirect data could be found [19].

Here, we exploited the development of an HFn-based nanoformulation of Everolimus (HEve) for the treatment of different subtypes of BC, regardless of their PI3K/AKT/mTOR activity status.

The aim of the present study was to assess HEve anticancer efficacy in comparison with free Eve in Eve-sensitive (i.e., BT474) and Eve-resistant (i.e., SKBR3, MDA-MB 231 and MCF-7) cell lines.

## 2. Materials and Methods 

### 2.1. H-Ferritin (HFn)

HFn were purchased from Molirom s.r.l. (Rome, Italy). HFn was labelled with fluoresceine isothiocyanate Isomer I (Sigma-Aldrich S.r.l., CAS Number: 3326-32-7, Milan, Italy) according to the manufacturer’s protocol (Sigma-Aldrich S.r.l, Milan, Italy) for interaction studies with BC cell lines.

### 2.2. HFn Loading with Eve 

HEve was prepared using the drug complexation with Cu(II) [15]. Everolimus powder (25 mg; Aurogene S.r.l., Rome, Italy; S1120-25MG) was previously solubilized in Ethanol (2.5 mL; 10 mg/mL). Eve (100 µg) was incubated for 10 min at RT with 10 mM CuSO_4_ obtaining a Cu(II)–Eve complex. The complexed drug was added to HFn (1 mg) and then incubated for 2 h at RT. HEve was separated from free Eve by a gel filtration to Zeba™ Spin Desalting Column (Thermo Fisher Scientific, Monza, Italy; Catalog Number: 89890). Protein content of HFn in HEve sample was assessed by Bradford assay (Thermo Fisher Scientific, Monza, Italy; Catalog Number: 23200), while the quantitative UPLC/MS-MS analysis determined the amount of encapsulated Eve.

### 2.3. Eve Quantification

Eve quantification was performed by an Ultra High Performance Liquid Chromatography (UHPLC) system equipped with a Triple Quadrupole Mass Spectrometer (6460 Agilent Technologies, Santa Clara, CA, USA). The instrument, operating in positive mode with electrospray ionization interface (ESI), was used to carry out MS/MS analysis (UHPLC-MS/MS). For the chromatographic conditions, a Zorbax Eclipse plus C18 2.1 × 50 mm 1.8 µm column was used. The chromatographic run time was 6 min.

To quantify HEve after the loading procedure, 80 μL of cold acetonitrile was added to 20 μL of HEve sample. Then, the mixture was vortexed and centrifuged (10 min, 14,000 rpm) in order to precipitate HFn. Fifty microliters of the supernatant was diluted with mobile phase. Afterwards, 100 μL of the sample with its internal standard (EveD4) was directly injected into the UHPLC-MS/MS system. A six-point calibration curve (62.5–2500 ng/mL) was used to quantify Eve encapsulated in HFn.

In order to obtain the precipitation solution, a mineral acid (37% HCl) was diluted in acetone. PBS samples were vortexed and incubated in an ice bath for 30 min and then centrifuged for 15 min at 14,000 rpm. Afterwards, the supernatant was dried under a weak flow of nitrogen (N_2_). Samples were then reconstituted in 100 μL of mobile phase prior to UHPLC-MS/MS analysis. To quantify drug release, the calibration curve was prepared in PBS and ranged between 5.0 and 50.0 ng/mL. 

For HEve quantification in cytoplasm, a solid phase extraction (SPE) procedure followed the protein precipitation. The residue was reconstituted with aqueous phase and loaded onto preconditioned cartridges. Then, 100 μL of mobile phase was used to inject the samples into the UHPLC-MS/MS system. The limit of detection (LOD) of the analytical method was set at 0.5 ng/mL. The calibration curve prepared in the matrix used throughout the study was linear between 1.0 and 50.0 ng/mL sample.

### 2.4. Transmission Electron Microscopy

A drop of HFn suspension was positioned on the Formvar net and dried at RT. Then, the net was stained with uranil-acetate 1% for 30 s at RT and dried over night at RT [15]. Samples were evaluated by Transmission Electron Microscopy (Tecnai Spirit, FEI, Hillsboro, OR, USA). Magnification 220,000× and 135,000×.

### 2.5. Kinetics of Spontaneous Eve Release In Vitro

HEve was collected in a dialysis device (Float-A-lyzer G2 Dialysis Device MWCO: 100 KD, SpectrumLABS, Compton, CA, USA) and kept in a PBS bath at 37 °C for seven days. At predetermined time points (15 min, 30 min, 1 h, 2 h, 3 h, 4 h, 5h, 6 h, 24 h, 48 h, 72 h, 96 h, 168 h), 5 mL of buffer were collected and replaced with fresh buffer in order to maintain sink condition. The amount of released drug in each collected sample was quantified by UPLC/MS-MS, as described above. 

### 2.6. Cell Cultures

SKBR3, MCF-7, and MDA-MB-231 cell lines were purchased by ATCC-LGC Standards and Caliper LifeSciences, respectively. BT474 was kindly provided by Dr. Libero Santarpia (IRCCS Humanitas Clinical and Research Center, Milano, Italy). 

BT474 and MCF-7 cells were cultured in DMEM Medium supplemented with 10% FBS, l-glutamine (2 mM), penicillin (50 UI/mL) and streptomycin (50 mg/mL). SKBR3 were cultured in DMEM/Ham’s F12 Medium supplemented with 10% FBS, l-glutamine (2 mM), penicillin (50 UI/mL) and streptomycin (50 mg/mL). MDA-MB 231 cells were cultured in MEM Medium supplemented with 10% FBS, l-glutamine (2 mM), penicillin (50 UI/mL), and streptomycin (50 mg/mL). All cell lines grow at 37 °C in humidified atmosphere containing 5% CO_2_ and were subcultured prior to confluence using trypsin/EDTA. Cell culture medium and chemicals for cell culture were purchased from Euroclone S.p.A.

### 2.7. TfR1 Expression

SKBR3, BT474, and MCF-7 cells (5 × 10^5^) were labelled in FACS tubes with anti-TfR1 antibody diluted 1:100 (1 µg/tube; CD71 Antibody (clone ICO-92), Thermo Fisher Scientific, Catalog Number #: MA1-7657) in blocking buffer (PBS, 2% Bovine Serum Albumin (BSA; Sigma-Aldrich S.r.l., Milan, Italy) and 2% goat serum (Euroclone S.p.A., Pero, Italy) for 30 min at RT and cells were washed three times with PBS. Then, cells were labelled with Alexa Fluor 488 goat anti-mouse secondary antibody diluted 1:200 (1 µL/tube; Thermo Fisher Scientific, Monza, Italy; Catalog Number #: A-11001) in blocking buffer for 30 min at RT and were washed thrice with PBS before analysis using CytoFLEX flow cytometer (Beckman Coulter, Cassina De’ Pecchi, Italy). 20,000 events were acquired, after gating on viable cells and on singlets. Cells immunodecorated with secondary antibody only were used to set the region of positivity.

### 2.8. In Plate-Cell Binding Assay at 37 °C

A total of 5 × 10^5^ cells was seeded on a six -well plate. The day after seeding, cells were incubated for 1 h at 37 °C in culture medium with 10 and 100 µg/mL of FITC-labelled HFn. After incubation, cells were washed thrice with PBS, collected from the plate with trypsin/EDTA and suspended in 0.5 mL of PBS before analysis using CytoFLEX flow cytometer (Beckman Coulter, Cassina De’ Pecchi, Italy). A total of 20,000 events was analyzed for each sample, after gating on viable cells and on singlets. The appropriate gates were set using a sample of untreated cells. 

### 2.9. HFn Internalization—Confocal Laser Scanning Microscopy

A total of 2 × 10^5^ cells was seeded on cover glass slides precoated with collagen in a six-well plate. The day after seeding, cells were treated with 100 μg/mL of FITC-labelled HFn for different time periods (15 min, 1, 3 and 24 h) at 37 °C to evaluate the intracellular fate of HFn. After incubations, cells were washed with PBS, fixed 5 min with 4% paraformaldehyde (Sigma-Aldrich S.r.l., Milan, Italy) and then permeabilized 5 min with 0.1% Triton X-100 (Sigma-Aldrich S.r.l., Milan, Italy). A blocking step was executed for 1 h at RT with a solution containing 2% BSA (Sigma-Aldrich S.r.l., Milan, Italy), 2% goat serum (Euroclone S.p.A., Pero, Italy) and 0.2 μg/mL DAPI (4′,6-diamino-2-phenylindole; Thermo Fisher Scientific, Monza, Italy). Confocal microscopy images were acquired with the Leica SP8 system equipped with laser excitation lines at 405, 488, 552, and 633 nm, using 63× magnification oil immersion lenses at 1024 × 1024 or 512 × 512 pixel resolution.

### 2.10. Cell Viability Assay

Cells were seeded on a 96-well well plate at the density of 4,000 cells cm^−1^ and the day after were incubated with different amounts of Eve or HEve (1, 10, 50 and 100 nM). After 72 h of treatment, cells were washed with PBS before incubation for 3 h at 37 °C with 0.1 mL of a stock solution of 3-(4,5-dim ethylthiazol-2-yl)-5-(3carboxymethoxyphenyl)-2-(4-sulfophenyl)-2H-tetrazolium (MTS) and phenazine ethosulfate (PES) previously diluted five times in phenol red-free medium (CellTiter 96^®^ AQueous One Solution Reagent; Promega, Madison, WI, USA; Catalog #: G5421). A microplate reader (BioTek, Milan, Italy) read absorbance using a testing wavelength of 490 nm and a reference wavelength of 620 nm. Untreated samples were used to normalize the results and expressed as means ± s.e.

### 2.11. Cell Death Assay

A total of 2 × 10^5^ cells was seeded on a 12-well plate and treated for 72 h in the presence of different amounts of Eve or HEve (10, 50, and 100 nM). Untreated cells represented negative control. At the end of incubation, cells were collected and washed three times with PBS. Then, cells were suspended in Binding Buffer and incubated for 5 min with 5 μL of Annexin V-PE-Cy5, according to Annexin V-PE-Cy5 Apoptosis Detection Kit manufacturer’s protocol (BioVision, Milpitas, CA, USA; Catalog #: K129-100). CytoFLEX flow cytometer (Beckman Coulter, Cassina De’ Pecchi, Italy) was used to acquire 20,000 events/sample. 

### 2.12. Quantification of Cytoplasmatic Accumulation of Eve

A total of 1 × 10^6^ cells was seeded on a six-well plate and incubated at 37 °C with Eve or HEve (10, 50 and 100 nM) for 24 h. At the end of incubation time, cells were collected with Trypsin/EDTA and centrifuged 5 min at 2000 rpm. Pellets were washed two times with PBS, suspended in 1 mL of Nuclei Extraction Buffer (10 mM Hepes, pH 7.4, 320 mM Sucrose, 5 mM MgCl, 1% Triton X-100) before incubation on ice for 10 min. Cytoplasm was separated by nuclei centrifuging the sample 5 min at 2000 rpm. The supernatant (cytoplasmatic fraction) was collected and processed for UPLC/MS-MS evaluation of Eve content.

## 3. Results

### 3.1. HFn Nanoparticles: Interaction with Tumor Cells and Internalization

In order to develop a nanodrug delivery system able to improve Eve penetration in BC cells, we have previously assessed the capability of HFn to target the MDA-MB 231 TNBC cell line [15]. Here, we demonstrated its targeting efficiency also in BT474, SKBR3, and MCF-7. This allowed us to evaluate the capability of HFn nanocages to interact with a panel of in vitro models of BC, using cell lines both sensitive (BT474) and resistant (SKBR3, MDA-MB 231, MCF-7) to Eve, according to classification reported by Hurvitz et al. [21]. Moreover, all the major BC subtypes have been represented since HER2-positive (i.e., BT474 and SKBR3), triple-negative (i.e., MDA-MB 231), and estrogen receptor positive (i.e., MCF-7) cell lines have been employed. All these cells display a good degree of Transferrin Receptor-1 (TfR1) expression, suggesting the positive role of HFn nanocages in improving drug internalization (Supplementary Figure S1) [15]. To assess the suitability of HFn as targeted delivery system for Eve, we have evaluated its interaction with the selected panel of BC cells performing a binding assay in physiological conditions (37 °C, 5% CO_2_).

**Figure 1:**
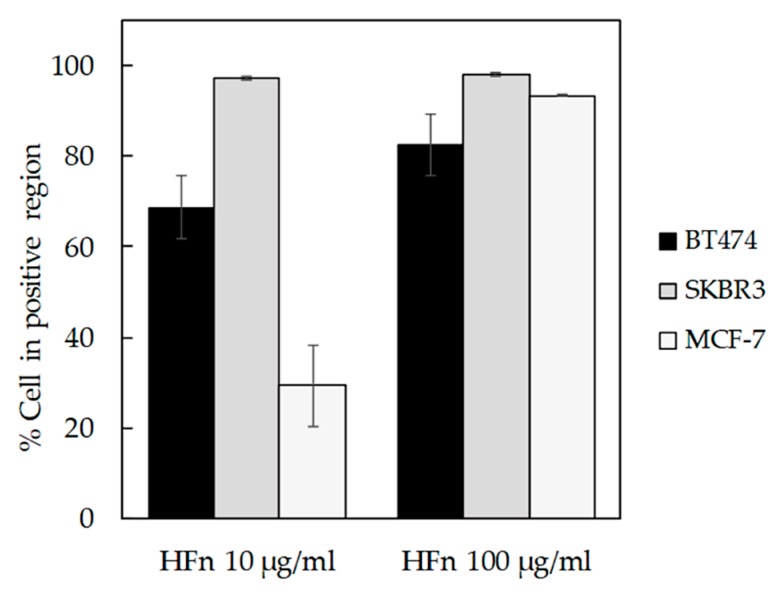
HFn recognition of BC cells. BT474, SKBR3, and MCF-7 cells were incubated for 1 h at 37 °C in complete cell culture medium with different amounts of FITC-labelled HFn nanoparticles (10 and 100 μg/mL). Then, cells were collected, washed, and processed for flow cytometry, using untreated cells to set the positive and the singlet gates. Reported values are the means ± s.e. (*n* = 3).

BT474, SKBR3, and MCF-7 cells have been incubated 1 h with increasing concentrations of fluoresceine isothiocyanate (FITC)-labelled HFn (10 or 100 μg/mL). Then, the binding percentage has been assessed by flow cytometry, demonstrating a good and dose-dependent capability of HFn to recognize all the tested cell lines (Figure 1, Supplementary Figures S2–S4), confirming the interaction’s data already published with MDA-MB 231 [15]. Moreover, the binding percentage at 10 µg/mL was assessed.

HFn is indirectly related to TfR1 expression, as expected when specific binding takes place. Indeed, in SKBR3 cells that display low TfR1 expression, there are few HFn binding sites per cell, so less HFn is necessary to saturate them, as demonstrated by SKBR3 results at 10 µg/mL (Supplementary Figure S4). Otherwise, in cells lines with higher TfR1 expression (i.e., BT474 and MCF-7), binding site saturation occurs at higher HFn dosages (Supplementary Figures S2 and S3). 

**Figure 2:**
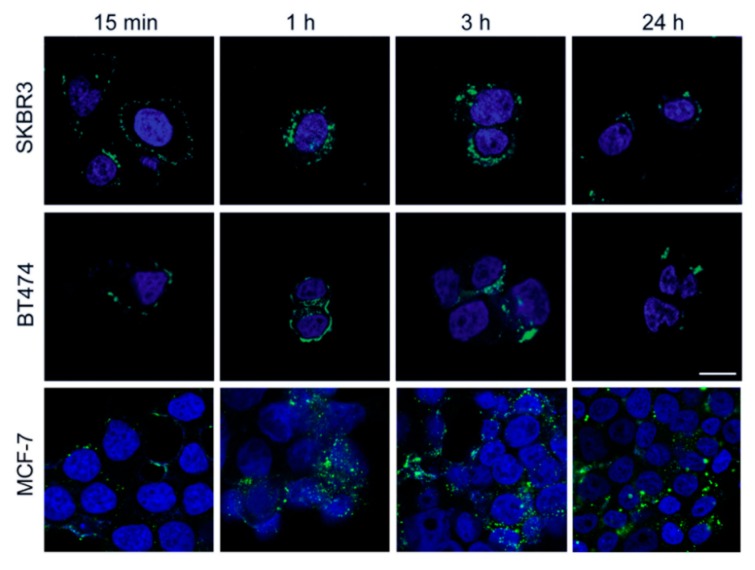
Localization of HFn. Confocal images of SKBR3, BT474, and MCF-7 cells incubated for 15 min, 1 h, 3 h, and 24 h at 37 °C in complete cell culture medium with FITC-labelled HFn nanoparticles (green; 100 μg/mL). Nuclei were stained with DAPI (blue). Scale bar = 10 µm.

Confocal laser scanning images of BC cells incubated with FITC-labelled HFn nanoparticles evidenced that a fast interaction with cellular membrane occurred, since FITC-labelled HFn was attached to the cell surface even after 15 min of incubation. In addition, FITC-labelled HFn was already internalized after 1 h of incubation and, after 3 h, the internalization process was almost complete. After 24 h, the fluorescence signal of FITC-labelled HFn decreased (Figure 2), in accordance with results from previous studies [17,20], suggesting that HFn nanocages were degraded or combined with native HFn, which is unlabelled.

### 3.2. Development of HEve Nanodrug

**Figure 3:**
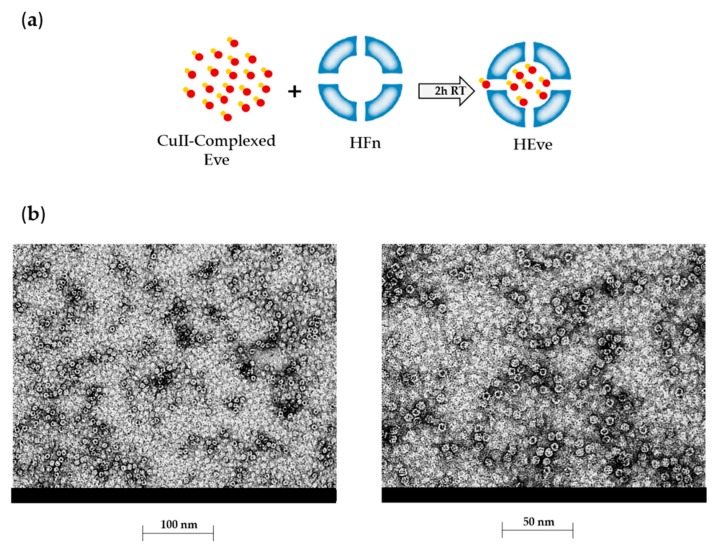
Development and characterization of HEve. (**a**) Schematic representation of the HFn loading strategy used for HEve production. After Everolimus precomplexation with Cu(II), the loading of HFn nanocage with Eve is a metal-driven process. Everolimus is represented in red, with Cu(II) in orange. (**b**) Electron microscopy images of Everolimus-loaded HFn nanoparticles obtained with the Cu(II) precomplexation strategy.

To set up the experimental conditions required for Eve incorporation into the HFn nanocages, we exploited HFn’s affinity towards metal ions to drive the uptake of Eve [22]. Eve was complexed with Cu(II) by incubation with 10 mM CuSO_4_. Then, the complexed drug was added to HFn and incubated to allow Cu(II)-driven incorporation of Eve in HFn as depicted in Figure 3a. Unloaded Eve was separated by HEve through gel filtration, and the amount of recovered HEve was determined by quantifying HFn and Eve by Bradford assay and by UHPLC-MS/MS, respectively. Results reported in Table 1 summarized the yields of HEve production with the drug-complexation strategy. Although the percentage of HFn recovered after the loading procedure is high (about 75%) and the protocol is very reproducible, only a little amount of Eve has been encapsulated inside the HFn nanocage, resulting in about 0.35 molecules of Eve in a single HFn cage. Despite the low encapsulation efficiency of Eve in our HFn nanocages, this is enough to exert a significant biological effect, as shown in the following paragraphs. However, we have investigated about the quality of HEve nanodrug. The structural integrity of HFn nanocages of HEve was analyzed by transmission electron microscopy (TEM). TEM images showed a homogenous suspension of cave sphere structures, demonstrating that the quaternary structure of HFn was preserved after Eve loading (Figure 3b). 

**Figure 4:**
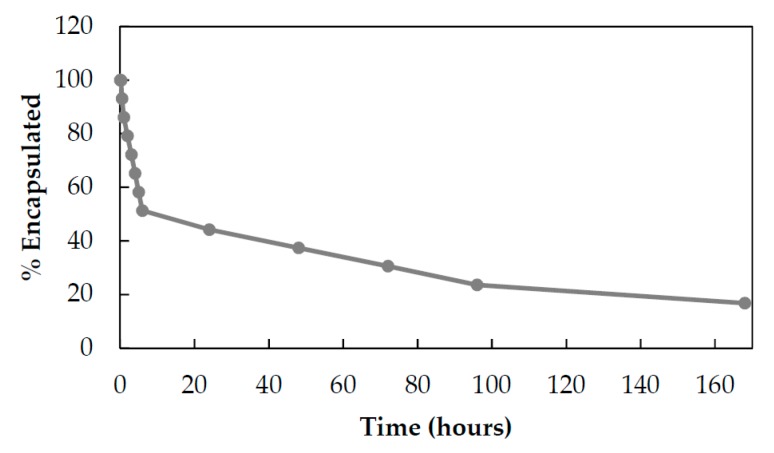
Drug release from HEve nanoparticles. The kinetics of Eve release from HFn nanocages was determined by UHPLC-MS/MS quantifying the amount of Eve released at 37 °C in PBS buffer.

Next, Eve release from HFn was analyzed in physiological conditions to assess the kinetic of release from HFn nanocage. This kinetic was estimated by dialyzing HEve at 37 °C in phosphate-buffered saline (PBS) and measuring Eve leakage from HFn by UHPLC-MS/MS quantification in the supernatants. Eve is released following first order kinetics with a constant release of drug (6–7% every hour) during the first 6 h. After 24 h, the kinetic slows down to 6–7% of release/day (Figure 4).

### 3.3. HEve Exhibits Enhanced Anticancer Activity in Tumor Cells

**Figure 5:**
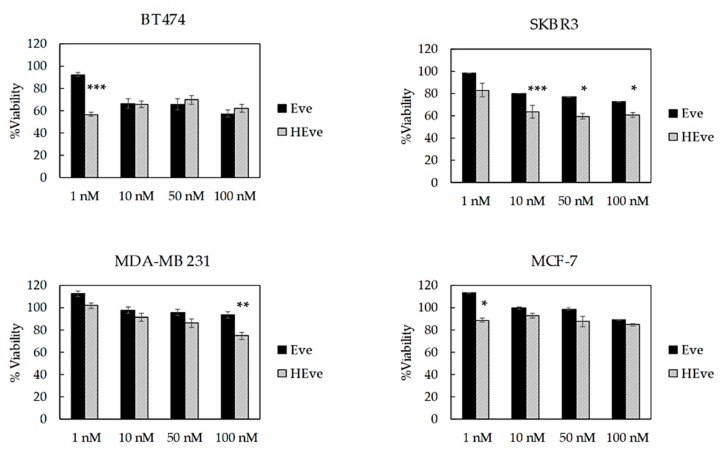
Viability of BC cells treated with Eve or HEve nanoparticles. BT474, SKBR3, MDA-MB 231, and MCF-7 cells were treated with 1, 10, 50, and 100 nM Eve or HEve for 72 h. Viability was assessed by measuring the conversion of MTS into formazan. Reported values are the mean of six replicates ± s.e., normalized to cell proliferation of untreated cells. Statistical significance of HEve vs. free drug, * *p* < 0.05; ** *p* < 0.005; *** *p* < 0.0005 (Student’s *t*-test).

To further investigate the quality of the HEve nanodrug, we have assessed its antitumor efficacy in comparison with Eve in a panel of TNBC, HER2, and ER positive cell lines. Cells have been treated with different amounts of Eve or HEve in a concentration range between 1 and 100 nM for 72 h (Figure 5) and the percentage of viable cells was assessed by MTS assay, normalizing treated samples toward untreated controls. BT474 cell viability was affected by treatment with 10, 50, and 100 nM Eve and HEve (Figure 5) in a similar manner. However, the Eve nanoformulation in HFn significantly increased the anticancer effect of Eve at 1 nM concentration, as evidenced in Figure 5, suggesting the potential role of nanoformulation in reducing drug-dosage in Eve-sensitive tumor cells. Furthermore, the nanoformulation in HFn displayed a positive contribution in Eve-resistant cell lines. Indeed, HEve exhibited an increase in antitumor activity as compared to Eve in SKBR3 cells (Figure 5), revealing a significant difference in the anticancer potential between the free and nanoformulated drug. Moreover, HEve was able to cause a notable reduction in MDA-MB 231 and MCF-7 cell viability, while free Eve did not affect viability of these cell lines (Figure 5). Although several evidences about the overall safety of the HFn nanocages have been already available in literature [15], we have assessed the effect of void HFn nanocages on BT474, SKBR3, and MCF-7 cells. MTS assay results reported in Supplementary Figure S5 confirmed that HFn does not affect BC cells’ viability. To exclude a toxic contribution of copper used for the encapsulation reaction, we performed MTS assays incubating cells with 1.5, 0.15, and 0.015 µM of copper. Results reported in supplementary Figure 6 clearly evidence that cell viability is not affected by copper in assay conditions. On the whole, viability results clearly evidenced the positive contribution of HFn nanoformulation in increasing the therapeutic potential of Eve both in sensitive and resistant cell lines.

### 3.4. The Treatment with HEve Determines Cell Apoptosis

**Figure 6:**
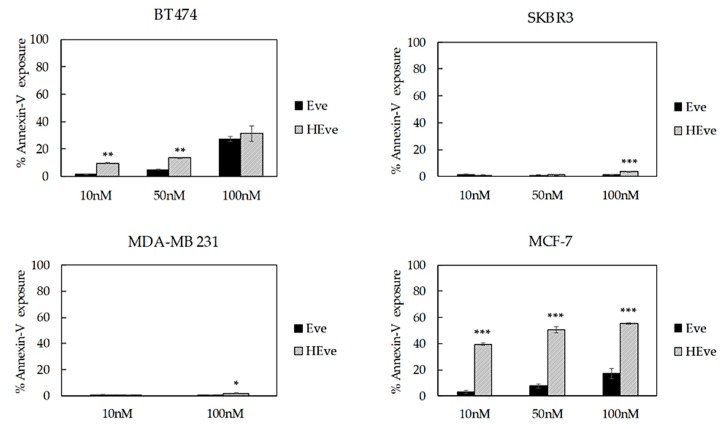
Cell death assay of BT474, SKBR3, MDA-MB 231, and MCF-7 cells treated with increasing concentrations of nanoformulated Eve (HEve; 10, 50, and 100 nM) or Eve for 72 h. Reported values are the mean of three replicates ± s.e., normalized to cell proliferation of untreated cells. * *p* < 0.005; ** *p* < 0.0005; *** *p* < 0.0001 (Student’s *t*-test).

Next, in order to determine if the reduced proliferation observed in HEve treated samples was associated with an increase in cell death, we have assessed the exposure to Annexin V, which is expected to only occur in apoptotic cells. BC cells were incubated for 72 h with Eve or HEve at different concentrations, including 10 nM, 50 nM, and 100 nM. Annexin V exposure was measured by flow cytometry using untreated cells to set the regions of positivity. The percentage of dead cells reported in Figure 6 demonstrated that HEve was much more effective than free Eve in inducing apoptosis at low concentrations in the sensitive cell line (i.e., BT474). At higher concentrations of Eve, differences between free or nanoformulated drug were smoothed, as expected, since we hypothesized that the positive contribution of nanoformulation is mainly due to a more efficient and rapid delivery of drug inside the cells.

Eve-resistant SKBR3 and MDA-MB 231 cells treated with low concentration of HEve did not show any significant increase in cell death in comparison with free drug, since the Eve dosage was probably too low to significantly affect cell death (Figure 6 and Figure S7). However, increasing drug concentrations up to 100 nM made the advantage of nanoformulated HEve in comparison to free Eve clear (Figure 6 and Figure S7). Instead, in the MCF-7 cell line (Figure 6), Eve-induced apoptosis is visible in test conditions. Therefore, the ability of nanoformulated Eve to induce cell death is prominent.

### 3.5. HFn Mediates Cytoplasmatic Delivery of Eve

To explain the increased efficacy of Eve upon HFn nanoformulation, we have hypothesized a positive role of HFn in mediating drug uptake, thus increasing the amount of payload released into the cytoplasmatic compartment. Therefore, we incubated BT474 cells for 24 h and 72 h with 10 or 100 nM Eve or HEve and measured the amount of drug accumulated in the cytoplasmatic compartment by UHPLC-MS/MS, in order to investigate whether HFn could play an active role in the cytoplasmatic delivery of Eve. Results reported in Table 2 show that HFn played a crucial role in improving Eve cytoplasmatic accumulation (Table 2). Indeed, in cells treated with free Eve, the amount of drug is below the detection limit, while in samples treated with 100 nM HEve, the drug is clearly detected in the cytoplasm.

## 4. Discussion

Eve’s FDA approval is limited to the treatment of hormone receptor-positive advanced BC in progression after aromatase inhibitor treatment, if associated to exemestane, following the findings from the BOLERO-2 trial [1]. However, controversial results have been reported with triple-negative BC in the adjuvant setting and, in one report, also in the neoadjuvant setting [2]. Also, in HER2-positive disease, adding Eve after occurrence of resistance to trastuzumab has demonstrated a relatively limited benefit, according to findings of the BOLERO-3 trial [23]. However, dysregulation of the PI3K/AKT/mTOR pathway is widely reported among BC cell subtypes, suggesting that there may be a window of opportunity for administration of Eve in BC, independent of the biological subtype [24]. Furthermore, the limited benefit of Eve, particularly in hormone receptor-negative BC, is counterbalanced by a high toxicity due to off-target actions of the drug, particularly grade 3–4 neutropenia and stomatitis [1,2]. Here, a novel nanodrug consisting of H-ferritin nanoparticles loaded with Eve was developed, tested, and compared to free Eve for in vitro efficacy on sensitive and resistant BC cell lines used as in vitro models of HER2^+^, triple-negative, and ER^+^ BC. Our study’s goal was to increase the spectrum of action of Eve by optimizing its cellular uptake and subcellular delivery by nanoformulation in HFn nanocages. HFn displays natural tumor homing thanks to its capability to specifically recognize cancer cells by exploiting the interaction with TfR1 [13,15,20,25]. This receptor is overexpressed in cancer cells as a result of their active iron metabolism [13].

Although the amount of Eve encapsulated in HFn nanocages is lower than observed with other drugs [13,20], its formulation in HFn nanocages increases the drug’s ability to inhibit BC cell proliferation thanks to its increased cellular uptake, probably due to TfR1-mediated internalization [13]. TfR1 is a key factor in iron homeostasis, together with transferrin. In healthy tissues, TfR1 is ubiquitously expressed at low levels, while in tumors it is overexpressed due to the highly active iron metabolism that characterizes this pathology. [25,26]. TfR1 is also able to bind and internalize circulating Ferritin by exploiting a specific interaction with the H-ferritin chain [25]. Therefore, the development of nanoformulated anticancer drugs using nanocages of H-ferritin is a reasonably good solution to improve drug effectiveness [14,15,19,20]. As confirmed by flow cytometry, all BC cell lines used here display TfR1 overexpression (Figure S1); therefore, there is good binding between HFn nanocages and cells (Figure 1). The HFn binding of BC cells drives rapid nanocage internalization, as evidenced in Figure 2.

Nanoformulation confers Eve with remarkable antiproliferative activity as compared to free Eve in all tested lines, making resistant cell lines sensitive to Eve (Figure 5). HEve showed a higher antiproliferative effect at 1 nM concentration and significantly improved cell death compared to free Eve. Similarly, by using HEve it is possible to reduce drug dosages, as evidenced in Figure 5 with BT474 cells, where the same antiproliferative effect could be obtained with 1 nM HEve or with 100 nM Eve. Antiproliferative results of HEve are full in accordance with cytotoxic activity observed by cell death assay. Here, HEve is more effective than Eve in inducing cell death in Eve-resistant cells (Figure 6 and Figure S7), confirming a role of nanoformulation in powering the antitumor activity of Eve. Although the 10–15% decrease in viability observed in luminal MCF-7 cells was expected, its coupling with a high induction of cell death needs to be better explained. The increased PI3K activity, resulting from a high PI3KCA gene copy number and activating PI3KCA point mutations found in MCF-7 cells, determines hyperactivation of AKT [24], which stimulates cell proliferation in different ways [27]. Here, the inhibition of mTOR-dependent proliferation due to Eve treatment is negligible in comparison to the stimulated proliferation induced by other AKT effectors and therefore explains the limited but significant reduction in viability observed upon Eve and HEve treatment [27]. However, the more pronounced effects observed in the cell death assay find an explanation in the molecular profile of this cell line. MCF-7 does not express caspase-3; it exploits alternative apoptotic mechanisms [28] that generally result in quicker cell death in comparison to caspase-3-expressing cells [29].

Triple-negative BC cell lines less frequently rely on the PI3K/AKT/mTOR pathway [24]. The findings of our study suggest that low doses of nanoformulated Eve allow the drug to be internalized at higher concentrations in the cytoplasm, thus exerting a relevant effect in cell lines such as MDA-MB 231. Furthermore, the results of the cell death assay performed on Eve-sensitive cell lines support HFn nanoformulation in order to reduce drug dosage.

Since all the tested BC cell lines display a significant increase in anticancer efficacy by HEve, UHPLC-MS/MS results demonstrate that it is due to the improved drug uptake following HFn nanoformulation. However, different responses to HEve could occur in other BC cell lines, since Eve uptake could be differently influenced by TfR1 expression. In addition, Eve sensitivity is a parameter to take into account. Further studies are needed to exactly disclose the incidence of these two features. However, our results support the therapeutic success of HEve in a relevant panel of BCs and the quick HFn uptake observed by confocal microscopy in all BC cell lines (Figure 2) suggests that the advantage due to delivery could be similarly effective in cells with different TfR1 expression levels.

Finally, few instances of Everolimus-loaded nanoparticles have been developed in the literature. These include gold nanoparticles decorated with CD44 and chitosan nanoparticles decorated with hyaluronic acid and CD44 [30,31,32]. However, these applications are related to other pathologies. Moreover, in the case of gold nanoparticles, an increase in drug efficacy upon nanoformulation was not documented. Only one work employed PLGA nanoparticles loaded with Eve to treat cancer, but in this case, efficacy was only achieved through codelivery with paclitaxel [33].

## 5. Conclusions

Here, a novel H-ferritin-based Eve nanoformulation has been developed, tested, and compared to free Eve on sensitive and resistant HER2^+^, triple-negative, and ER^+^ BC cell lines. By optimizing Eve’s subcellular delivery, we have improved its antitumor activity.

## 6. Patents

Number 102018000009959 (Italian patent, deposit date 31/10/2018).

## Figures and Tables

**Table 1:** Summary of yields of HFn recovery after Everolimus encapsulation.

Encapsulation Strategy	% HFn Recovery	Eve/HFn
Drug complexation with CuII	75.23 ± 3.93	0.35 ± 0.07

Data were generated from 26 samples of HEve.

**Table 2:** Cytoplasmic extract. Reported values are the mean of three samples ± s.e. Statistical significance of HEve vs. free drug. * *p* < 0.05; ** *p* < 0.0005 (Student’s *t*-test).

Sample	Conc (ng/mL)	Time (hours)
Eve 1 0 nM	<0.5	24
Eve 100 nM	<0.5	24
HEve 10 nM	<0.5	24
* HEve 100 nM	1.94 ± 0.36	24
Eve 10 nM	<0.5	72
Eve 100 nM	<0.5	72
HEve 10 nM	<0.5	72
** HEve 100 nM	3.74 ± 0.08	72

## Supplementary Materials

The following are available online at https://www.mdpi.com/1999-4923/11/8/384/s1, Figure S1. Expression of TfR1. Figure S2. Binding assay in MCF-7 cells. Figure S3. Binding assay in BT474 cells. Figure S4. Binding assay in SKBR3 cells. Figure S5. Safety of void HFn nanocages. Figure S6. Safety of copper used for encapsulation. Figure S7. Cell death assay of SKBR3 and MDA-MB 231 cells treated with increasing concentration of nanoformulated Eve or Eve for 72 h.
