# Supplementary material for: Everolimus Nanoformulation in Biological Nanoparticles Increases Drug Responsiveness in Resistant and Low-Responsive Breast Cancer Cell Lines
Source: Pharmaceutics. 2019 Aug 2;11(8):384. doi: 10.3390/pharmaceutics11080384 (PMC6723888; doi:10.3390/pharmaceutics11080384)
Supplement: Supplementary file 1 [file pharmaceutics-11-00384-s001.pdf]

pharmaceutics

Supplementary Materials: Everolimus Nanoformulation in Biological Nanoparticles Increases Drug Responsiveness in Resistant and Low-Responsive Breast Cancer Cell Lines

Arianna Bonizzi, Marta Truffi, Marta Sevieri, Raffaele Allevi, Leopoldo Sitia, Roberta Ottria, Luca Sorrentino, Cristina Sottani, Sara Negri, Elena Grignani, Serena Mazzucchelli and Fabio Corsi

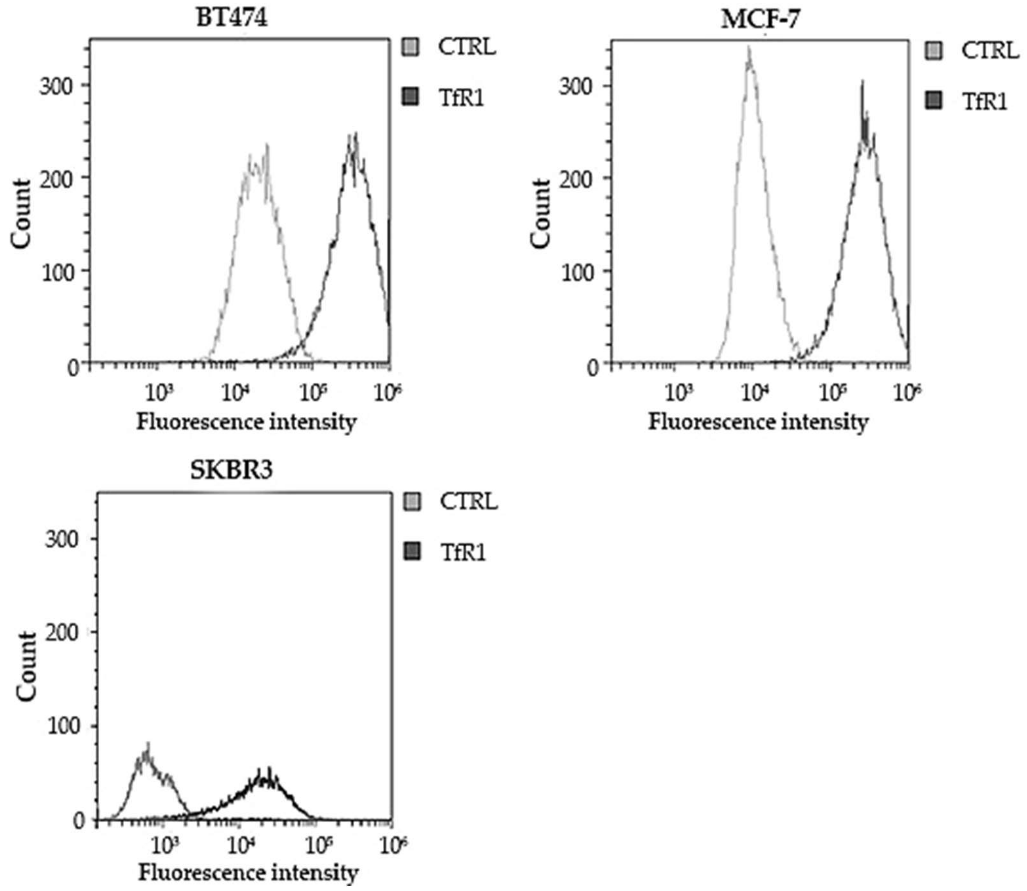

Figure S1. Expression of TfR1. Flow cytometry analysis of membrane TfR1 expression. HER2- positive (i.e. BT474 and SKBR3) and Estrogen Receptor positive (i. e. MCF-7) breast cell lines have been tested assessing their membrane TfR1 expression. Cells immunodecorated with the anti-mouse secondary antibody conjugated with AlexaFluor 488 were used to set the gate on viable cells, on singlets and the region of positivity.

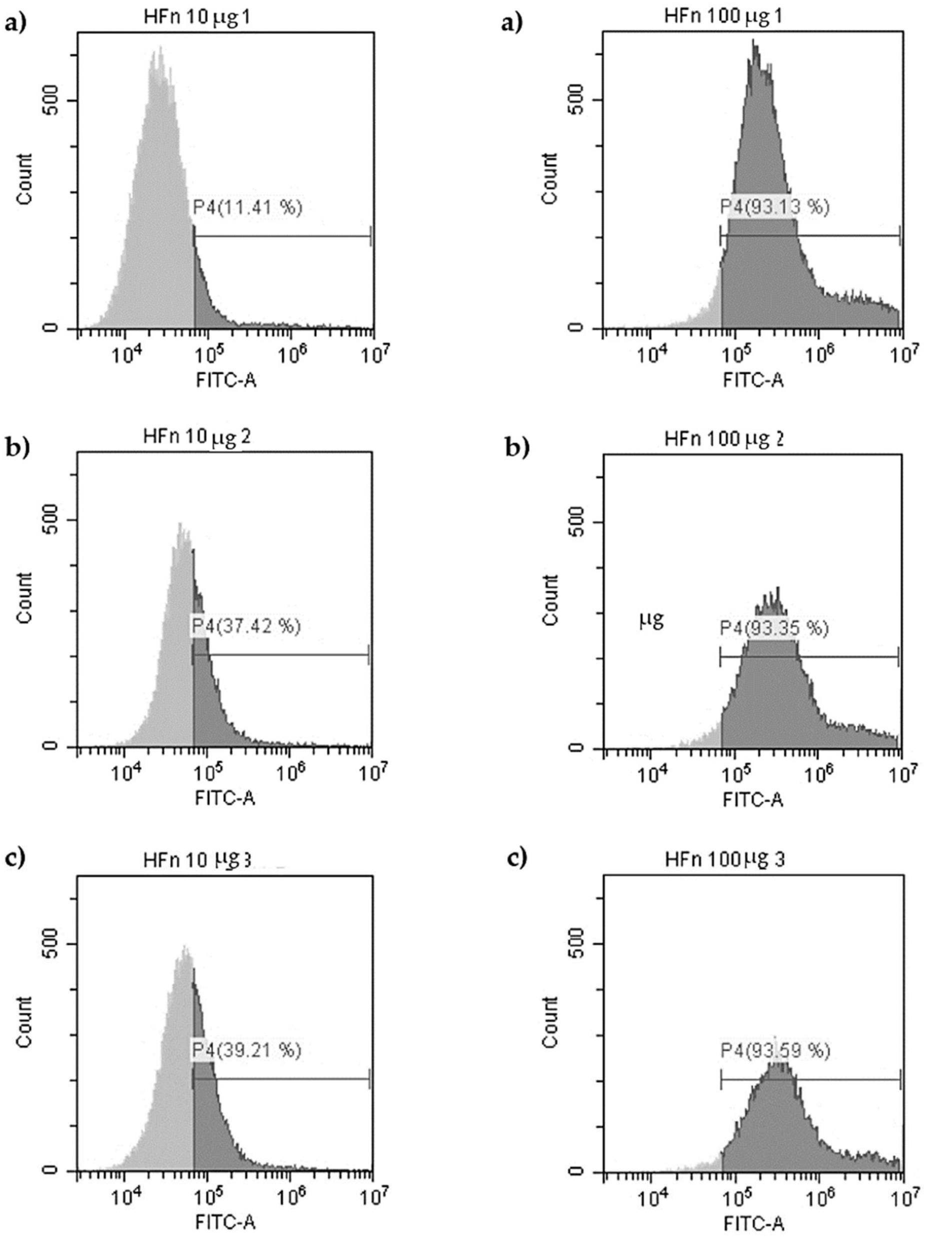

Figure S2. Binding assay in MCF-7 cells. Flow cytometry analysis of binding assay performed after incubation of MCF-7 cells with 10 and 100 µg/mL of FITC-labelled HFn. Untreated cells have been used to set positivity region. Analysis has been performed after gating on singlets. Panel a, b and c reported results of different experimental replicates.

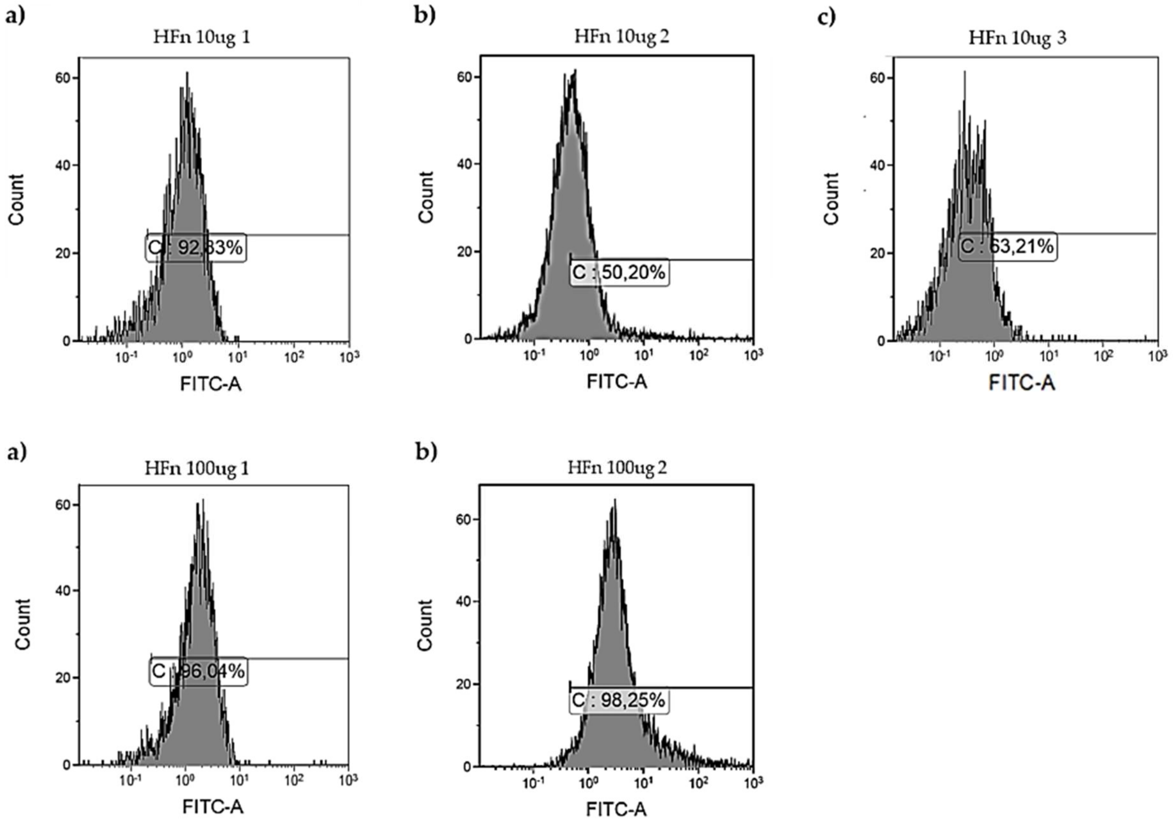

Figure S3. Binding assay in BT474 cells. Flow cytometry analysis of binding assay performed after incubation of BT474 cells with 10 and 100 μg/mL of FITC-labelled HFn. Untreated cells have been used to set positivity region. Analysis has been performed after gating on singlets. Panel a, b and c reported results of different experimental replicates.

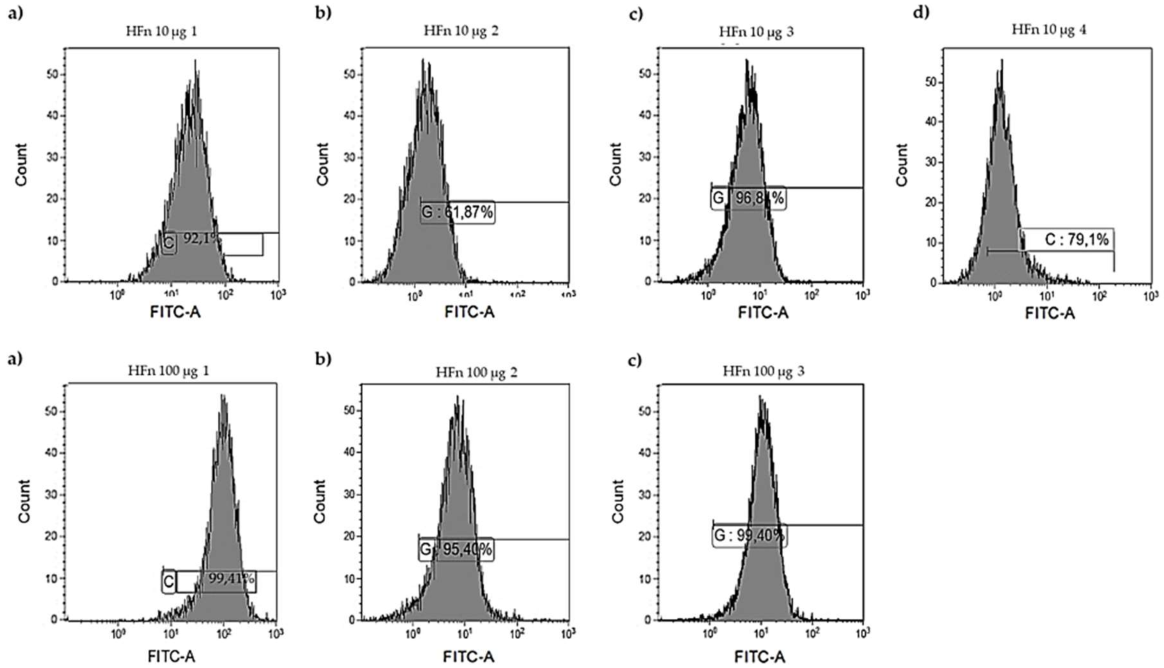

Figure S4. Binding assay in SKBR3 cells. Flow cytometry analysis of binding assay performed after incubation of SKBR3 cells with 10 and 100 μg/mL of FITC-labelled HFn. Untreated cells have been used to set positivity region. Analysis has been performed after gating on singlets. Panel a, b, c and d reported results of different experimental replicates.

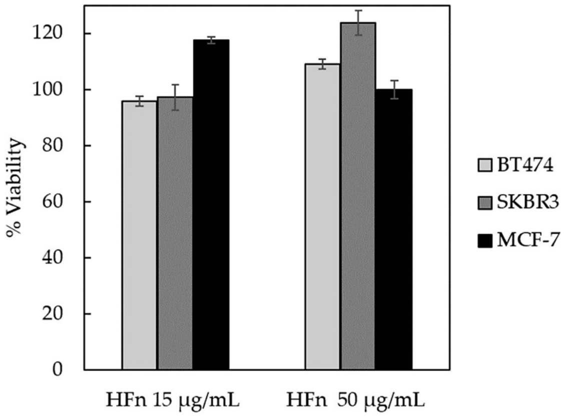

Figure S5. Safety of void HFn nanocages. BT474, SKBR3 and MCF-7 cells have been treated for 72 h with 15 and 50 µg/mL of void HFn. Viability has been assessed by MTS assay. Reported values are the mean of six replicates ± s.e., normalized on cell proliferation of untreated cells, respectively. Statistical significance vs. untreated cells, *P < 0.05; **P < 0.005; ***P < 0.0005 (Student's t-test).

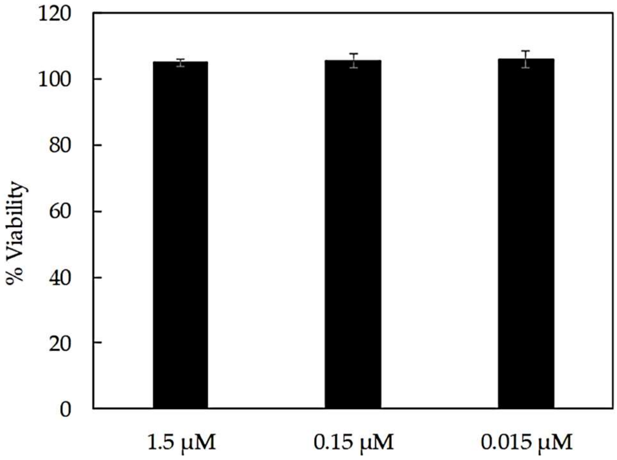

Figure S6. Safety of copper used for encapsulation. MCF-7 cells treated with 1.5, 0.15 and 0.015 µM of copper for 72 h have been used to demonstrate the safety of copper used for Eve nanoformulation. Viability was assessed by measuring the conversion of MTS into formazan. Reported values are the mean of six replicates ± s.e., normalized on cell proliferation of untreated cells, respectively. Statistical significance vs. untreated cells, *P < 0.05; **P < 0.005; ***P < 0.0005 (Student's t-test).

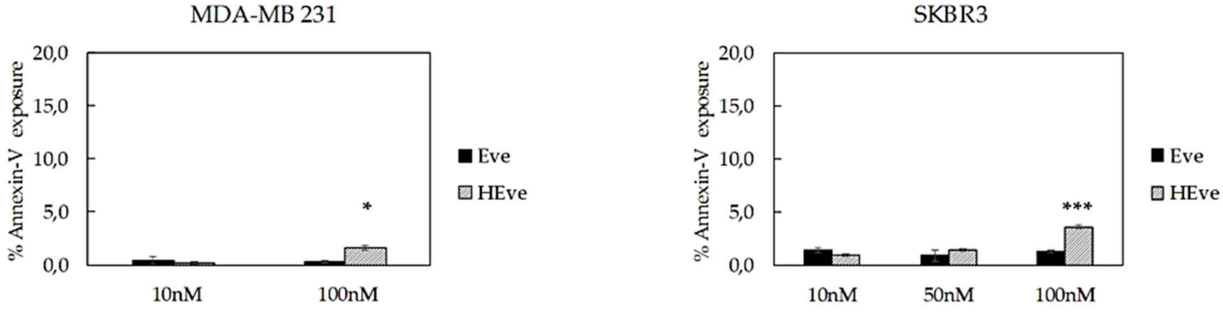

Figure S7. Cell death assay of SKBR3 and MDA-MB 231 cells treated with increasing concentration of nanoformulated Eve or Eve for 72h. Reported values are the mean of three replicates ± s.e., normalized on cell proliferation of untreated cells, respectively. Statistical significance of HEve vs. free drug; *P < 0.005; ***P < 0.0001 (Student's t-test).
